# Leveraging collective action and environmental literacy to address complex sustainability challenges

**DOI:** 10.1007/s13280-022-01764-6

**Published:** 2022-08-09

**Authors:** Nicole M. Ardoin, Alison W. Bowers, Mele Wheaton

**Affiliations:** 1 Emmett Interdisciplinary Program in Environment and Resources, Graduate School of Education, and Woods Institute for the Environment, Stanford University, 233 Littlefield Hall, Stanford, CA 94305 USA; 2 Social Ecology Lab, Graduate School of Education and Woods Institute for the Environment, Stanford University, 233 Littlefield Hall, Stanford, CA 94305 USA; 3 Emmett Interdisciplinary Program in Environment and Resources, School of Earth, Energy and Environmental Sciences, Stanford University, 473 Via Ortega, Suite 226, Stanford, CA 94305 USA

**Keywords:** Collective action, Community, Environmental literacy, Social movements, Sustainability

## Abstract

Developing and enhancing societal capacity to understand, debate elements of, and take actionable steps toward a sustainable future at a scale beyond the individual are critical when addressing sustainability challenges such as climate change, resource scarcity, biodiversity loss, and zoonotic disease. Although mounting evidence exists for how to facilitate individual action to address sustainability challenges, there is less understanding of how to foster collective action in this realm. To support research and practice promoting collective action to address sustainability issues, we define the term “collective environmental literacy” by delineating four key potent aspects: scale, dynamic processes, shared resources, and synergy. Building on existing collective constructs and thought, we highlight areas where researchers, practitioners, and policymakers can support individuals and communities as they come together to identify, develop, and implement solutions to wicked problems. We close by discussing limitations of this work and future directions in studying collective environmental literacy.

## Introduction

For socio-ecologically intertwined issues—such as climate change, land conversion, biodiversity loss, resource scarcity, and zoonotic diseases—and their associated multi-decadal timeframes, individual action is necessary, yet not sufficient, for systemic, sustained change (Amel et al. 2017; Bodin 2017; Niemiec et al. 2020; Spitzer and Fraser 2020). Instead, collective action, or individuals working together toward a common good, is essential for achieving the scope and scale of solutions to current sustainability challenges. To support communities as they engage in policy and action for socio-environmental change, communicators, land managers, policymakers, and other practitioners need an understanding of how communities coalesce and leverage their shared knowledge, skills, connections, and experiences.

Engagement efforts, such as those grounded in behavior-change approaches or community-based social marketing initiatives, that address socio-environmental issues have often emphasized individuals as the pathway to change. Such efforts address a range of domains including, but not limited to, residential energy use, personal transportation choices, and workplace recycling efforts, often doing so in a stepwise fashion, envisioning each setting or suite of behaviors as discrete spheres of action and influence (Heimlich and Ardoin 2008; McKenzie-Mohr 2011). In this way, specific actions are treated incrementally and linearly, considering first the individual barriers to be removed and then the motivations to be activated (and, sometimes, sustained; Monroe 2003; Gifford et al. 2011). Once each behavior is successfully instantiated, the next barrier is then addressed. Proceeding methodically from one action to the next, such initiatives often quite successfully alter a series of actions or group of related behaviors (at least initially) by addressing them incrementally, one at a time (Byerly et al. 2018). Following this aspirational logic chain, many resources have been channeled into such programs under the assumption that, by raising awareness and knowledge, such information, communication, and educational outreach efforts will shift attitudes and behaviors to an extent that, ultimately, mass-scale change will follow. (See discussion in Wals et al. 2014.)

Numerous studies have demonstrated, however, that challenges arise with these stepwise approaches, particularly with regard to their ability to address complex issues and persist over time (Heimlich and Ardoin 2008; Wals et al. 2014). Such approaches place a tremendous—and unrealistic—burden on individuals, ignoring key aspects not only of behavioral science but also of social science more broadly, including the view that humans exist nested within socio-ecological systems and, thus, are most successful at achieving lasting change when it is meaningful, relevant, and undertaken within a supportive context (Swim et al. 2011; Feola 2015). Individualized approaches often require multiple steps or nudges (Byerly et al. 2018), or ongoing reminders to retain their salience (Stern et al. 2008). Because of the emphasis on decontextualized action, such approaches can miss, ignore, obfuscate, or minimize the importance of the bigger picture, which includes the sociocultural, biophysical, and political economic contexts (Ardoin 2006; Amel et al. 2017). Although the tightly trained focus on small, actionable steps and reliance on individual willpower may help in initially achieving success with initial habit formation (Carden and Wood 2018), it becomes questionable in terms of bringing about a wave of transformation on larger scales in the longer term. For those decontextualized actions to persist, they require continued prompting, constancy, and support in the social and biophysical context (Schultz 2014; Manfredo et al. 2016; Wood and Rünger 2016).

Less common in practice are theoretically based initiatives that embrace the holistic nature of the human experience, which occurs within complex systems spanning time and space in a multidimensional, weblike fashion (Bronfenbrenner 1979; Rogoff 2003; Barron 2006; DeCaro and Stokes 2008; Gould et al. 2019; Hovardas 2020). These systems-thinking approaches, while varying across disciplines and epistemological perspectives, envision human experiences, including learning and behavior, as occurring within a milieu that include the social, political, cultural, and historical contexts (Rogoff 2003; Roth and Lee 2007; Swim et al. 2011; Gordon 2019). In such a view, people’s everyday practices continuously reflect and grow out of past learning and experiences, not only at the individual, but also at the collective level (Lave 1991; Gutiérrez and Rogoff 2003; Nasir et al. 2020; Ardoin and Heimlich 2021). The multidimensional context in which we exist—including the broader temporal and spatial ecosystem—both facilitates and constrains our actions.

Scholars across diverse areas of study discuss the need for and power of collective thought and action, using various conceptual frames, models, and terms, such as collective action, behavior, impact, and intelligence; collaborative governance; communities of practice; crowdsourcing; and social movement theory; among many others (Table 1). These scholars acknowledge and explore the influence of our multidimensional context on collective thought and action. In this paper, we explore the elements and processes that constitute *collective environmental literacy*. We draw on the vast, relevant literature and, in so doing, we attempt to invoke the power of the collective: by reviewing and synthesizing ideas from a variety of fields, we strive to leverage existing constructs and perspectives that explore notions of the “collective” (see Table 1 for a summary of constructs and theories reviewed to develop our working definition of collective environmental literacy). A primary goal of this paper is to dialogue with other researchers and practitioners working in this arena who are eager to uncover and further explore related avenues.

**Table 1:** Summary of constructs and theories, derived from theoretical and empirical literature, influencing the development and conceptualization of collective environmental literacy

Constructs/theories	Sample citations	Literature-derived concepts informing collective environmental literacy key principles/definition
Collective constructs		
Collective action	Amel et al. (2017), Bamberg et al. (2015), Chan (2016), Graham et al. (2019), Groulx et al. (2017), Jagers et al. (2020), Jost et al. (2017), Niemiec et al. (2020), Ostrom (1990, 2000, 2010), van Zomeren et al. (2008)	Power of the collective, synergy Antecedents/factors/frameworks of effective collective efforts Role of shared resources, including social capital and knowledge Issues of power dynamics
Collective behavior	Blumer (1971), Gordon (2019), Granovetter (1978), Park (1927), Smelser (2011/1962), Turner and Killian (1987)	
Collective efficacy	Bandura (2000), Sampson et al. (1997), Thaker et al. (2019)	
Collective impact	Kania and Kramer (2011)	
Collective intelligence	Lévy and Bononno (1997), Weschsler (1971), Woolley et al. (2010)	
Collective memory	Fanta et al. (2019), Halbwachs (1992/1941/1952/), Olick (1999)	
Collective literacies		
Collective environmental literacy	Bey et al. (2020)	Contribution of collective environmental literacy to healthier, more resilient, and more equitable communities
Collective health literacy, community health literacy, critical health literacy, distributed health literacy, population health literacy, public health literacy	Barry et al. (2013), Chinn (2011), Edwards et al. (2015), Freedman et al. (2009), Guzys et al. (2015), Papen (2009), Sørensen et al. (2012)	Acknowledgment of learning as a social process Benefits of collectivity for each community Value of diverse perspectives and community strengths Engagement with existing groups and efforts Literacy as distributed throughout a social network
Collective information literacy	Bruce and Chesterton (2002), Lloyd (2005), Martin and Steinkuehler (2010)	Applying a collective lens to literacy Value of collaboration and shared practices
Collective science literacy; community science literacy	Feinstein (2018), Lee and Roth (2003), National Academies of Sciences, Engineering, and Medicine (2016), Roth (2003) Roth and Barton (2004), Roth and Lee (2002, 2004) Schoerning (2018), Spitzer and Fraser (2020)	Acknowledgment of literacy at a collective scale Role of social skills, social determinants, and social support in learning; social learning Literacy as asset rather than risk factor Literacy as process rather than static outcome Synergy of individual and collective literacies
Group-related/collaborative constructs		
Co-production of knowledge	Djenontin and Meadow (2018), Hill et al. (2020), Norström et al. (2020)	Context-based Interactive and iterative Ongoing Goal-oriented, problem/issue-focused
Collaboration–coordination continuum	Prager (2015), Sadoff and Grey (2005)	Continuum of levels of collaboration Level of collaboration impacted by socio-ecological factors
Collaborative governance	Ansell and Gash (2008), Bodin (2017), Emerson et al. (2012)	Value derived from collaboration Iterative, dynamic process Role of context, power, trust, shared understanding, and commitment
Communities of practice	Lave and Wenger (1991), Wenger and Snyder (2000)	Shared knowledge and practices, collective learning Problem-solving efficiency Self-selected members Shared interest, concern, or passion
Community environmental education	Aguilar (2018), Aguilar et al. (2015), Krasny et al. (2017)	Learning as a social process Contribution of collective efforts to community building
Community-based participatory research, citizen science, community-based monitoring participatory research	Berkes et al. (2007), Fernandez-Gimenez et al. (2008), Jull et al. (2017), McKinley et al. (2017)	Shared knowledge, including Indigenous knowledge Advancing science Encouraging action Acknowledging and engaging diversity within the community Building trust and community
Community resilience	Aldrich and Meyer (2015), Berkes and Ross (2013), Cutter et al. (2008), Koliou et al. (2020), Magis (2010), Sharifi (2016) Sherrieb et al. (2010)	Measures for the same community-scale construct can be quite different Value of including multiple dimensions (e.g., physical, economic, and social) Social capital impacts communities in many ways
Crowdsourcing	Assis Neto and Santos (2018), Brabham (2013), Karachiwalla and Pinkow (2021), Massung et al. (2013), Surowiecki (2005)	Power of crowds/groups, more than the sum of the parts Value of bottom-up approach Impact of groups on creativity
Cultural-historical activity theory (CHAT)	Engeström (2001), Roth and Lee (2007)	Learning occurs in social, cultural, and historical context Value of group/social learning
Funds of knowledge	Barton and Tan (2009), Cruz et al. (2018), González et al. (2006)	Value of tapping into group-held historical and cultural knowledge
Indigenous knowledge	Agrawal (1995), Briggs (2005), Briggs and Sharp (2004), Gadgil et al. (1993), Hill et al. (2020), Mistry and Berardi (2016), Pawilen (2021)	Building on strengths of local communities that have been previously ignored and overlooked Value of tapping into historical memory and local knowledge
Social capital	Adger (2003), Adler and Kwon (2002), Ostrom and Ahn (2009), Putnam (2020)	Social networks and social structures as critical resources for collective action
Social learning	Bandura (1977), Ensor and Harvey (2015), Reed et al. (2010)	Learning occurs in a social context Value of shared knowledge, co-creation of knowledge, and diverse stakeholders
Social movement (theory)	McAdam (2017), McAdam and Boudet (2012)	Role of political system and institutional structure in collective action, and trust in those systems and structures
Social identity	Brieger (2019), Mackay et al. (2021), Masson and Fritsche (2021), Reicher et al. (2010), Scheepers and Ellemers (2019), Stets and Burke (2000)	Connection between the individual and group Group factors as antecedents of collective action
Trans/interdisciplinarity	Jörg (2011), Klein (1990), Knapp et al. (2019), Schipper et al. (2021)	Complex problems require collaboration among different fields and ways of thinking

First, we present a formal definition of collective environmental literacy. Next, we briefly review the dominant view of environmental literacy at the individual level and, in support of a collective take on environmental literacy, we examine various collective constructs. We then delve more deeply into the definition of collective environmental literacy by outlining four key aspects: scale, dynamic processes, shared resources, and synergy. We conclude by providing suggestions for future directions in studying collective environmental literacy.

## Defining collective environmental literacy

Decades of research in political science, economics, anthropology, sociology, psychology, and the learning sciences, among other fields (Chawla and Cushing 2007; Ostrom 2009; Sawyer 2014; Bamberg et al. 2015; Chan 2016; Jost et al. 2017) repeatedly demonstrates the effectiveness, and indeed necessity of, collective action when addressing problems that are inherently social in nature. Yet theoretical frameworks and empirical documentation emphasize that such collective activities rarely arise spontaneously and, when they do, are a result of preconditions that have sown fertile ground (van Zomeren et al. 2008; Duncan 2018). Persistent and effective collective action then requires scaffolding in the form of institutional, sociocultural, and political economic structure that provides ongoing support. To facilitate discussions of how to effectively support collective action around sustainability issues, we suggest the concept of “collective environmental literacy.” We conceptualize collective environmental literacy as more than collective action; rather, we suggest that the term encapsulates *action* along with its various supporting structures and resources. Additionally, we employ the word “literacy” as it connotes learning, intention, and the idea that knowledge, skills, attitudes, and behaviors can be enhanced iteratively over time. By using “literacy,” we strive to highlight the efforts, often unseen, that lead to effective collective action in communities. We draw on scholarship in science and health education, areas that have begun over the past two decades to theorize about related areas of collective science literacy (Roth and Lee 2002, 2004; Lee and Roth 2003; Feinstein 2018) and health literacy (Freedman et al. 2009; Papen 2009; Chinn 2011; Guzys et al. 2015). Although these evolving constructs lack consensus definitions, they illuminate affordances and constraints that exist when conceptualizing collective environmental literacy (National Academies of Sciences, Engineering, and Medicine [NASEM] 2016).

**Fig. 1:**
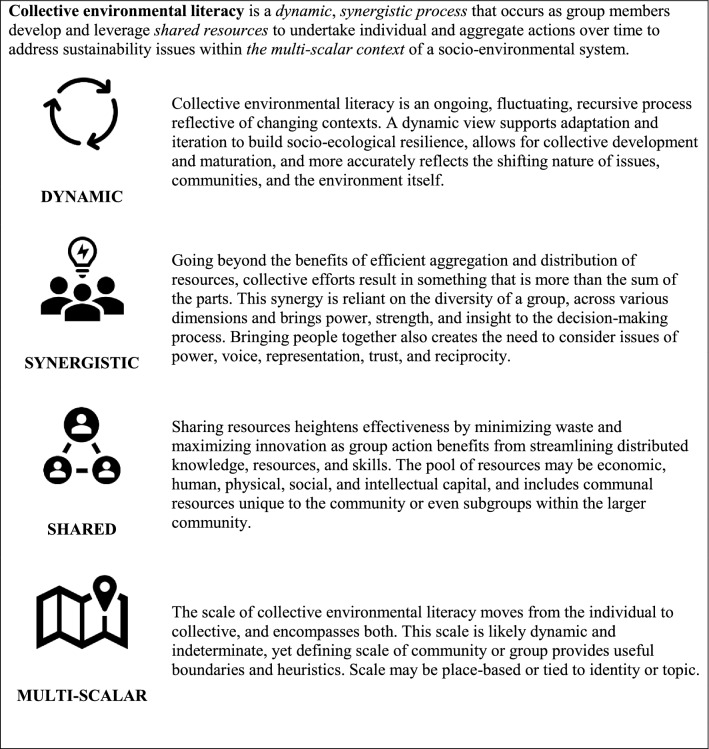
Key elements of collective environmental literacy

Some of the key necessary—but not sufficient—conditions that facilitate aligned, collective actions include a common body of decision-making information; shared attitudes, values, and beliefs toward a motivating issue or concern; and efficacy skills that facilitate change-making (Sturmer and Simon 2004; van Zomeren et al. 2008; Jagers et al. 2020). In addition, other contextual factors are essential, such as trust, reciprocity, collective efficacy, and communication among group members and societal-level facilitators, such as social norms, institutions, and technology (Bandura 2000; Ostrom 2010; McAdam and Boudet 2012; Jagers et al. 2020). Taken together, we term this body of knowledge, dispositions, skills, and the context in which they flourish *collective environmental literacy*. More formally, we define collective environmental literacy as: *a dynamic, synergistic process that occurs as group members develop and leverage shared resources to undertake individual and aggregate actions over time to address sustainability issues within the multi-scalar context of a socio-environmental system* (Fig. 1).

## Environmental literacy: Historically individual, increasingly collective

Over the past five decades, the term “environmental literacy” has come into increasingly frequent use. Breaking from the traditional association of “literacy” with reading and writing in formal school contexts, environmental literacy emphasizes associations with character and behavior, often in the form of responsible environmental stewardship (Roth 1992).^1^ Such perspectives define the concept as including affective (attitudinal), cognitive (knowledge-based), and behavioral domains, emphasizing that environmental literacy is both a *process* and *outcome* that develops, builds, and morphs over time (Hollweg et al. 2011; Wheaton et al. 2018; Clark et al. 2020).

The emphasis on defining, measuring, and developing interventions to bring about environmental literacy has primarily remained at the individual scale, as evidenced by frequent descriptions of an environmentally literate *person* (Roth 1992; Hollweg et al. 2011 among others) rather than *community* or *community member.* In most understandings, discussions, and manifestations of environmental literacy, the implicit assumption remains that the unit of action, intervention, and therefore analysis occurs at the individual level. Yet instinctively and perhaps by nature, community members often seek information and, as a result, take action collectively, sharing what some scholars call “the hive mind” or “group mind,” relying on each other for distributed knowledge, expertise, motivation, and support (Surowiecki 2005; Sunstein 2008; Sloman and Fernbach 2017; Paul 2021).

As with the proverbial elephant (Saxe, n.d.), each person, household, or neighborhood group may understand or “see” a different part of an issue or challenge, bring a novel understanding to the table, and have a certain perspective or skill to contribute. Although some environmental literacy discussions allude to a collective lens (e.g., Hollweg et al. 2011; Ardoin et al. 2013; Wheaton et al. 2018; Bey et al. 2020), defining, developing frameworks, and creating measures to assess the efficacy of such collective-scale sustainability-related endeavors has remained elusive.^2^ Looking to related fields and disciplines—such as ecosystem theory, epidemiology and public health, sociology, network theory, and urban planning, among others—can provide insight, theoretical frames, and empirical examples to assist in such conceptualizations (McAdam and Boudet 2012; National Research Council 2015) (See Table 1 for an overview of some of the many areas of study that informed our conceptualization of collective environmental literacy).

## Seeking the essence of the collective: Looking to and learning from others

The social sciences have long focused on “the kinds of activities engaged in by sizable but loosely organized groups of people” (Turner et al. 2020, para. 1) and addressed various collective constructs, such as collective behavior, action, intelligence, and memory (Table 1). Although related constructs in both the social and natural sciences—such as communities of practice (Wenger and Snyder 2000), collaborative governance (Ansell and Gash 2008; Emerson et al. 2012), and the collaboration–coordination continuum (Sadoff and Grey 2005; Prager 2015), as well as those from social movement theory and related areas (McAdam and Boudet 2012; de Moor and Wahlström 2019)—lack the word “collective” in name, they too leverage the benefits of collectivity. A central tenet connects all of these areas: powerful processes, actions, and outcomes can arise when individuals coalesce around a common purpose or cause. This notion of a dynamic, potent force transcending the individual to enhance the efficacy of outcomes motivates the application of a collective lens to the environmental literacy concept.

Dating to the 1800s, discussions of *collective behavior* have explored connections to social order, structures, and norms (Park 1927; Smelser 2011/1962; Turner and Killian 1987). Initially, the focus emphasized spontaneous, often violent crowd behaviors, such as riots, mobs, and rebellions. More contemporarily, sociologists, political scientists, and others who study social movements and collective behaviors acknowledge that such phenomena may take many forms, including those occurring in natural ecosystems, such as ant colonies, bird flocks, and even the human brain (Gordon 2019). In sociology, *collective action* represents a paradigm shift highlighting coordinated, purposeful pro-social movements, while de-emphasizing aroused emotions and crowd behavior (Miller 2014). In political science, Ostrom’s (1990, 2000, 2010) theory of collective action in the context of the management of shared resources extends the concept’s reach to economics and other fields. In education and the learning sciences, social learning and sociocultural theories tap into the idea of learning as a social-cognitive-cultural endeavor (Vygotsky 1980; Lave and Wenger 1991; Tudge and Winterhoff 1993; Rogoff 2003; Reed et al. 2010).

Collective action, specifically, and collective constructs, generally, have found their way into the research and practice in the fields of conservation, natural resources, and environmental management. Collective action theory has been applied in a range of settings and scenarios, including agriculture (Mills et al. 2011), invasive species management (Marshall et al. 2016; Sullivan et al. 2017; Lubeck et al. 2019; Clarke et al. 2021), fire management (Canadas et al. 2016; Charnley et al. 2020), habitat conservation (Raymond 2006; Niemiec et al. 2020), and water governance (Lopez-Gunn 2003; Baldwin et al. 2018), among others. Frameworks and methods that emphasize other collective-related ideas—like collaboration, co-production, and group learning—are also ubiquitous in natural resource and environmental management. These constructs include community-based conservation (DeCaro and Stokes 2008; Niemiec et al. 2016), community natural resource management (Kellert et al. 2000; Dale et al. 2020), collaboration/coordination (Sadoff and Grey 2005; Prager 2015), polycentricity (Galaz et al. 2012; Heikkila et al. 2018), knowledge co-production (Armitage et al. 2011; Singh et al. 2021), and social learning (Reed et al. 2010; Hovardas 2020). Many writings on collective efforts in the social sciences broadly, and applied in the area of environment specifically, provide insights into collective action’s necessary preconditions, which prove invaluable to further defining and later operationalizing collective environmental literacy.

## Unpacking the definition of collective environmental literacy: Anchoring principles

As described, we propose the following working definition of collective environmental literacy drawing on our analysis of related literatures and informed by scholarly and professional experience in the sustainability and conservation fields: *a dynamic, synergistic process that occurs as group members develop and leverage shared resources to undertake individual and aggregate actions over time to address sustainability issues within the multi-scalar context of a socio-environmental system* (Fig. 1). This definition centers on four core, intertwined ideas: the *scale* of the group involved; the *dynamic nature* of the process; *shared resources* brought by, available to, and needed by the group; and the *synergy* that arises from group interaction.

### Multi-scalar

When transitioning from the focus on individual to collective actions—and, herein, principles of environmental literacy—the most obvious and primary requisite shift is one of scale. Yet, moving to a collective scale does not mean abandoning action at the individual scale; rather, success at the collective level is intrinsically tied to what occurs at an individual level. Such collective-scale impacts leverage the power of the hive, harnessing people’s willingness, ability, and motivation to take action alongside others, share their ideas and resources to build collective ideas and resources, contribute to making a difference in an impactful way, and participate communally in pro-social activities.

Collective environmental literacy is likely dynamic in its orientation to scale, incorporating place-based notions, such as ecoregional or community-level environmental literacy (with an emphasis on geographic boundaries). On the other hand, it may encapsulate environmental literacy of a group or organization united by a common identity (e.g., organizational membership) or cause (e.g., old-growth forests, coastal protection), rather than solely or even primarily by geography. Although shifting scales can make measuring collective environmental literacy more difficult, dynamic levels may be a benefit when addressing planetary boundary issues such as climate change, biodiversity, and ocean acidification (Galaz et al. 2012). Some scholars have called for a polycentric approach to these large-scale issues in response to a perceived failure of global-wide, top-down solutions (Ostrom 2010, 2012; Jordan et al. 2018). Conceptualizing and consequently supporting collective environmental literacy at multiple scales can facilitate such desired polycentricity.

### Dynamic

Rather than representing a static outcome, environmental literacy is a dynamic process that is fluctuating and complex, reflective of iterative interactions among community members, whose discussions and negotiations reflect the changing context of sustainability issues.^3^ Such open-minded processes allow for, and indeed welcome, adaptation in a way that builds social-ecological resilience (Berkes and Jolly 2002; Adger et al. 2005; Berkes 2007). Additionally, this dynamism allows for collective development and maturation, supporting community growth in collective knowledge, attitudes, skills, and actions via new experiences, interactions, and efforts (Berkman et al. 2010). With this mindset, and within a sociocultural perspective, collective environmental literacy evolves through drawing on and contributing to the community’s funds of knowledge (González et al. 2006). Movement and actions within and among groups impact collective literacy, as members share knowledge and other resources, shifting individuals and the group in the course of their shared practices (Samerski 2019).

### Shared

In a collective mode, effectiveness is heightened as shared resources are streamlined, waste is minimized, and innovation maximized. Rather than each group member developing individual expertise in every matter of concern, the shared knowledge, skills, and behaviors can be distributed, pursued, and amplified among group members efficiently and effectively, with collective literacy emerging from the process of pooling diverse forms of capital and aggregating resources. This perspective builds on ideas of social capital as a collective good (Ostrom 1990; Putnam 2020), wherein relationships of trust and reciprocity are both inputs and outcomes (Pretty and Ward 2001). The shared resources then catalyze and sustain action as they are reassembled and coalesced at the group level for collective impact.

The pooled resources—likely vast—may include, but are not limited to, physical and human resources, funding, time, energy, and space and place (physical or digital). Shared resources may also include forms of theorized capital, such as intellectual and social (Putnam 2020). Also of note is the recognition that these resources extend far beyond information and knowledge. Of particular interest when building collective environmental literacy are resources previously ignored or overlooked by those in power in prior sustainability efforts. For example, collective environmental literacy can draw strength from shared resources unique to the community or even subgroups within the larger community. Discussions of Indigenous knowledge (Gadgil et al. 1993) and funds of knowledge (González et al. 2006; Cruz et al. 2018) suggest critical, shared resources that highlight strengths of an individual community and its members. Another dimension of shared resources relates to the strength of institutional connections, such as the benefits that accrue from leveraging the collective knowledge, expertise, and resources of organizational collaborators working in adjacent areas to further and amplify each other’s impact (Wojcik et al. 2021).

### Synergistic

Finally, given the inherent complexities related to defining, deploying, implementing, and measuring these dynamic, at-times ephemeral processes, resources, and outcomes at a collective scale, working in such a manner must be clearly advantageous to pressing sustainability issues at hand. Numerous related constructs and approaches from a range of fields emphasize the benefits of diverse collaboration to collective thought and action, including improved solutions, more effective and fair processes, and more socioculturally just outcomes (Klein 1990; Jörg 2011; Wenger and Snyder 2000; Djenontin and Meadow 2018). These benefits go beyond efficient aggregation and distribution of resources, invoking an almost magical quality that defines synergy, resulting in robust processes and outcomes that are more than the sum of the parts.

This synergy relies on the diversity of a group across various dimensions, bringing power, strength, and insight to a decision-making process (Bear and Woolley 2011; Curşeu and Pluut 2013; Freeman and Huang 2015; Lu et al. 2017; Bendor and Page 2019). Individuals are limited not only to singular knowledge-perspectives and skillsets, but also to their own experiences, which influence their self-affirming viewpoints and tendencies to seek out confirmatory information for existing beliefs (Kahan et al. 2011). Although the coming together of those from different racial, cultural, social, and economic backgrounds facilitates a collective literacy process that draws on a wider range of resources and equips a gestalt, it also sets up the need to consider issues of power, privilege, voice, and representation (Bäckstrand 2006) and the role of social capital, leading to questions related to trust and reciprocity in effective collectives (Pretty and Ward 2001; Folke et al. 2005).

## Leveraging the ‘Hive’: Proceeding with collective environmental literacy

This paper presents one conceptualization of collective environmental literacy, with the understanding that numerous ways exist to envision its definition, formation, deployment, and measurement. Characterized by a collective effort, such literacies at scale offer a way to imagine, measure, and support the synergy that occurs when the emphasis moves from an individual to a larger whole. By expanding the scale and focusing on shared responsibility among actors at the systems level, opportunities arise for inspiring and enabling a broader contribution to a sustainable future. These evolving notions serve to invite ongoing conversation, both in research and practice, about how to enact our collective responsibility toward, as well as vision of, a thriving future.

Emerging from the many discussions of shared and collaborative efforts to address socio-environmental issues, our conceptualization of collective environmental literacy is a first step toward supporting communities as they work to identify, address, and solve sustainability problems. We urge continued discussions on this topic, with the goal of understanding the concept of collective environmental literacy, how to measure it, and the implications of this work for practitioners. The conceptual roots of collective environmental literacy reach into countless fields of study and, as such, a transdisciplinary approach, which includes an eye toward practice, is necessary to fully capture and maximize the tremendous amount of knowledge, wisdom, and experience around this topic. Specifically, next steps to evolve the concept include engaging sustainability researchers and practitioners in discussions of the saliency of the presented definition of collective environmental literacy. These discussions include verifying the completeness of the definition and ensuring a thorough review of relevant research: Are parts of the definition missing or unclear? What are the “blank, blind, bald, and bright spots” in the literature (Reid 2019 p. 158)? Additionally, recognizing and leveraging literacy at a collective scale most certainly is not unique to environmental work, nor is adopting literacy-related language to conceptualize and measure process outcomes, although the former has consistently proven more challenging. Moreover, although we (the authors) appreciate the connotations and structures gained by using a literacy framework, we struggle with whether “environmental literacy” is the most appropriate and useful term for the conceptualizations as described herein; we, thus, welcome lively discussions about the need for new terminology.

Even at this early stage of conceptualization, this work has implications for practitioners. For scientists, communicators, policymakers, land managers, and other professionals desiring to work with communities to address sustainability issues, a primary take-away message concerns the holistic nature of what is needed for effective collective action in the environmental realm. Many previous efforts have focused on conveying information and, while a lack of knowledge and awareness may be a barrier to action in some cases, the need for a more holistic lens is increasingly clear. This move beyond an individually focused, information-deficit model is essential for effective impact (Bolderdijk et al. 2013; van der Linden 2014; Geiger et al. 2019). The concept of collective environmental literacy suggests a role for developing shared resources that can foster effective collective action. When working with communities, a critical early step includes some form of needs assessment—a systematic, in-depth process that allows for meaningfully gauging gaps in shared resources required to tackle sustainability issues (Braus 2011). Following this initial, evaluative step, an understanding of the components of collective environmental literacy, as outlined in this paper, can be used to guide the development of interventions to support communities in their efforts to address those issues.

Growing discussion of collective literacy constructs, and related areas, suggests researchers, practitioners, and policymakers working in pro-social areas recognize and value collective efforts, despite the need for clearer definitions and effective measures. This definitional and measurement work, in both research and practice, is not easy. The ever-changing, dynamic contexts in which collective environmental literacy exists make defining the concept a moving target, compounded by a need to draw upon work in countless, often distinct academic fields of study. Furthermore, the hard-to-see, inner workings of collective constructs make measurement difficult. Yet, the “power of the hive” is intriguing, as the synergism that arises from communities working in an aligned manner toward a unified vision suggests a potency and wave of motivated action essential to coalescing and leveraging individual goodwill, harnessing its power and potential toward effective sustainability solutions.

## Footnotes

1 See Stables and Bishop’s (2001) idea of defining environmental literacy by viewing the environment as “text.”

2 The climate change education literature also includes a nascent, but growing, discussion of collective-lens thinking and literacy. See, for example, Waldron et al. (2019), Mochizuki and Bryan (2015), and Kopnina (2016).

3 This conceptualization is similar to how some scholars describe collective health literacy (Berkman et al., 2010; Mårtensson and Hensing, 2012).
